# Investigating the binding affinity, molecular dynamics, and ADMET properties of curcumin-IONPs as a mucoadhesive bioavailable oral treatment for iron deficiency anemia

**DOI:** 10.1038/s41598-024-72577-8

**Published:** 2024-09-25

**Authors:** M. Yasser Alsedfy, A. A. Ebnalwaled, Mona Moustafa, Alaa Hassan Said

**Affiliations:** 1 Electronics and Nano Devices Lab, Faculty of Science, South Valley University, Qena, 83523 Egypt; 2 Physics Department, Faculty of Science, Minia University, Minya, Egypt; 3 Department of Radiology, Faculty of Applied Health Sciences, Sphinx University, New Assiut, Egypt

**Keywords:** Iron deficiency anemia, Iron oxide nanoparticles, Molecular docking, Dynamic simulation, Curcumin, Biophysics, Biotechnology, Computational biology and bioinformatics, Drug discovery

## Abstract

Iron deficiency anemia (IDA) is a common health issue, and researchers are interested in overcoming it. Nanotechnology green synthesis is one of the recent approaches to making efficient drugs. In this study, we modeled curcumin-coated iron oxide nanoparticles (cur-IONPs) to study their predicted toxicity and drug-likeness properties, then to investigate mucoadhesive behavior by docking cur-IONPs with two main mucin proteins in gastrointestinal tract (GIT) mucosa (muc 5AC and muc 2). Furthermore, the stability of cur-IONPs/protein complexes was assessed by molecular dynamics. Our in-silico studies results showed that cur-IONPs were predicted to be potential candidates to treat IDA due to its mucoadhesive properties, which could enhance the bioavailability, time residency, and iron absorbance through GIT, in addition to its high safety profile with high drug-likeness properties and oral bioavailability. Finally, molecular dynamic simulation studies revealed stable complexes supporting strength docking studies. Our results focus on the high importance of in-silico drug design studies; however, they need to be supported with in vitro and in vivo studies to reveal the efficacy, toxicity, and bioavailability of cur-IONPs.

## Introduction

Iron deficiency anemia (IDA) is the most common cause worldwide, mainly acquired from nutritional sources^1^. According to WHO (2019), it estimates that in 2019, the global prevalence of anemia in children aged 6–59 months was around 40%^2^. Iron supplementation are still preferred cost-effective strategies to face iron deficiency anemia, which increases the challenges of developing suitable iron fortificants regarding acceptability and bioavailability^3^. From this point, nanotechnology is a golden approach to design functional drugs with high efficacy and satisfying bioavailability.

Recently, Iron oxide nanoparticles (IONPs) have gained interest in various diagnostic and therapeutic nanomedicine applications, rising from their long retention, biocompatibility, biodegradability, and low toxicity. One of the recent nanotechnology methods to prepare nanoparticles is “green synthesis”, which helps reduce their toxicity and functionalize their surfaces. Green synthesis methods are preferred for simplicity, cost-effectiveness, non-chemical wasting, availability, non-toxicity, and safety.

Curcumin, a nutraceutical polyphenol compound derived from *Curcuma longa*, has received considerable attention due to its anti-cancer^4^, anti-microbial, anti-inflammatory^5^, and antidepressant activity^6^.

Loading curcumin on IONPs has been reported in many biomedical studies for various purposes. Encapsulating IONPs with curcumin improved the drug's bioavailability and biodistribution. A recent study assessing the multidose effect of cur-IONPs on male BALB/c mice reported that cur-IONPs levels remained high in the liver even ten days after the last injection, which means a slow clearance rate from the body^7^. Some studies reported that coating IONPs with mucoadhesive agents could increase the residency time in GIT, increasing iron absorption and improving therapeutic performance by triggered release^8,9^.

As known, mucin proteins are playing a crucial role in mucous forming in gastrointestinal tract (GIT), muc 5ac and muc2 are the most dominant types in GIT, this entails their specific impacts on oral drug bioavailability, from this point some studies investigated enhancing mucoadhesion to the muc2 and muc 5ac rich intestinal mucus layer as a strategy to prolong gastrointestinal retention time and increase bioavailability of some drug^10^.

There is a noticed lack in oral bioavailability studies of cur-IONPs. Therefore, we will use various approaches of in-silico drug assessment studies, including docking with two main kinds of mucin protein in the gastrointestinal tract to assess the mucoadhesive behavior of cur-IONPs. Moreover, the oral bioavailability assessment and ADMET studies were performed. Then, a molecular dynamic simulation of our molecule was conducted to investigate the interaction between cur-IONPs with Mucin 5AC (muc 5ac) and mucin 2 (muc 2).

## Materials and methods

### Molecular docking

#### Ligand preparation

**Fig. 1:**
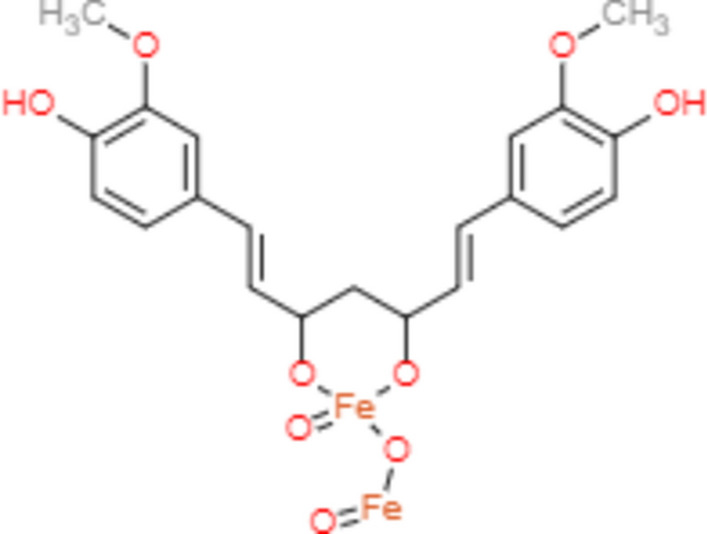
The chemical structure of the modeled cur-IONPs.

The chemical structure of curcumin was obtained from the PubChem database, and then it was modified using ChemDraw software to model cur-IONPs after revising the literature to specify the site of interaction between curcumin and IONPs during the coating process. The interaction occurs through making bonds between the divalent metal atom and the diketone groups of curcumin (Fig. 1)^11^. The modeled cur-IONPs were saved as a PDB file to be optimized and energetically minimized using molecular operating environment (MOE) 2014 software to study its affinity as a ligand to muc 5ac, and muc2 proteins.

#### Receptor preparation

The crystal structures of our chosen proteins were obtained from the protein data bank, adding hydrogen atoms, identifying the active sites for each protein, and selecting the site on which the docking will take place. Good-quality resolution protein structures were obtained from PDB. 1.70 Å, and 1.50 Å for Muc 5 AC, and Muc 2, respectively, considered a good quality for docking studies.

Molecular operation environments software (MOE) was used to perform docking and obtain scoring calculations. As known, the best scores of RMSD values should be near 2 Å. Regarding the energy scores, values around -7 kcal/mol are the best. RMSD and E scores are often used as a standard for validating molecular docking results.

#### ADMET studies

ADMET characteristics of drugs (e.g., Absorption, Distribution, Metabolism, Excretion, and Toxicology) are critical concepts to be assessed in drug pharmacokinetics. The ADMET analysis of the designed nanoparticle was performed using SwissADME (http://www.swissadme.ch) and ProTox-III webserver https://tox.charite.de/protox3/. Therefore, the structure's SMILE code of our cur-IONPs model was submitted to both servers, and the results were further analyzed.

#### Dynamic simulation studies

The MD simulations of this study were conducted for cur-IONPs/protein complexes, and the cabs-flex server was used to get RMSFs (root mean square fluctuations) through https://biocomp.chem.uw.edu.pl/CABSflex2/index. While IMODS server (https://imods.iqfr.csic.es/) was employed to determine the deformability and rigidity of our complexes depending on (normal mode analysis) NMA, which provides deformability, B-factors, eigenvalues, and variance plot.

## Results

### Molecular docking

**Fig. 2:**
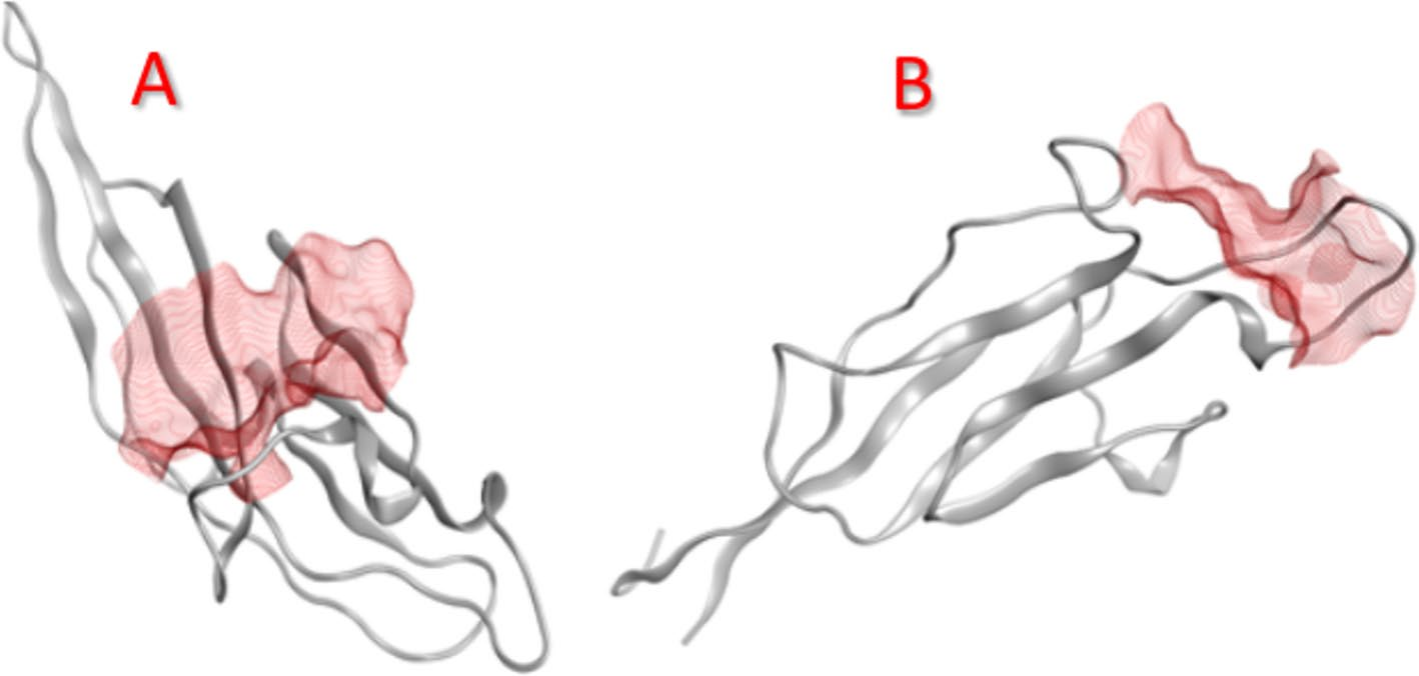
The active site of (**A**) muc 5AC and (**B**) muc 2 as specified by MOE 2014.

Firstly, we had to specify the active site position and residues sequence for each of our selected proteins. Table 1 and Fig. 2 show residues sequences and active site positions for each protein, respectively. The research results indicate that it is important to specify the active site residues of a protein before performing molecular docking. Specifying the active site residues allows the docking algorithm to generate poses that are more likely to be biologically relevant. In summary, defining the active site residues before docking, which helps ensure the generated poses, are more likely to represent the true binding mode, improving the overall reliability and accuracy of the docking results. This step is crucial in structure-based drug design and virtual screening applications^12–14^.

**Table 1:** Active site residues for each protein, which will act as receptor binding sites for ligands, specified using MOE built-in algorithms.

Protein	Code in PDB	Sites	Residues
Mucin 5AC (stomach)	8oV0	Site 1	1:(TRP3529 GLN3568 CYS3569 ARG3570 PRO3575 GLU3576 VAL3577 SER3578 ILE3579 GLU3580 VAL3586 GLN3587 CYS3588 SER3589)
Mucin 2 (intestine)	8ck2	Site 1	1:(PRO1314 SER1315 ASP1320 ASN1367 GLU1368 GLN1370 PHE1371 GLY1372 ASN1373 GLY1374 PRO1375 PHE1376 GLY1377 LEU1378)

### Proteins-ligands docking

**Fig. 3:**
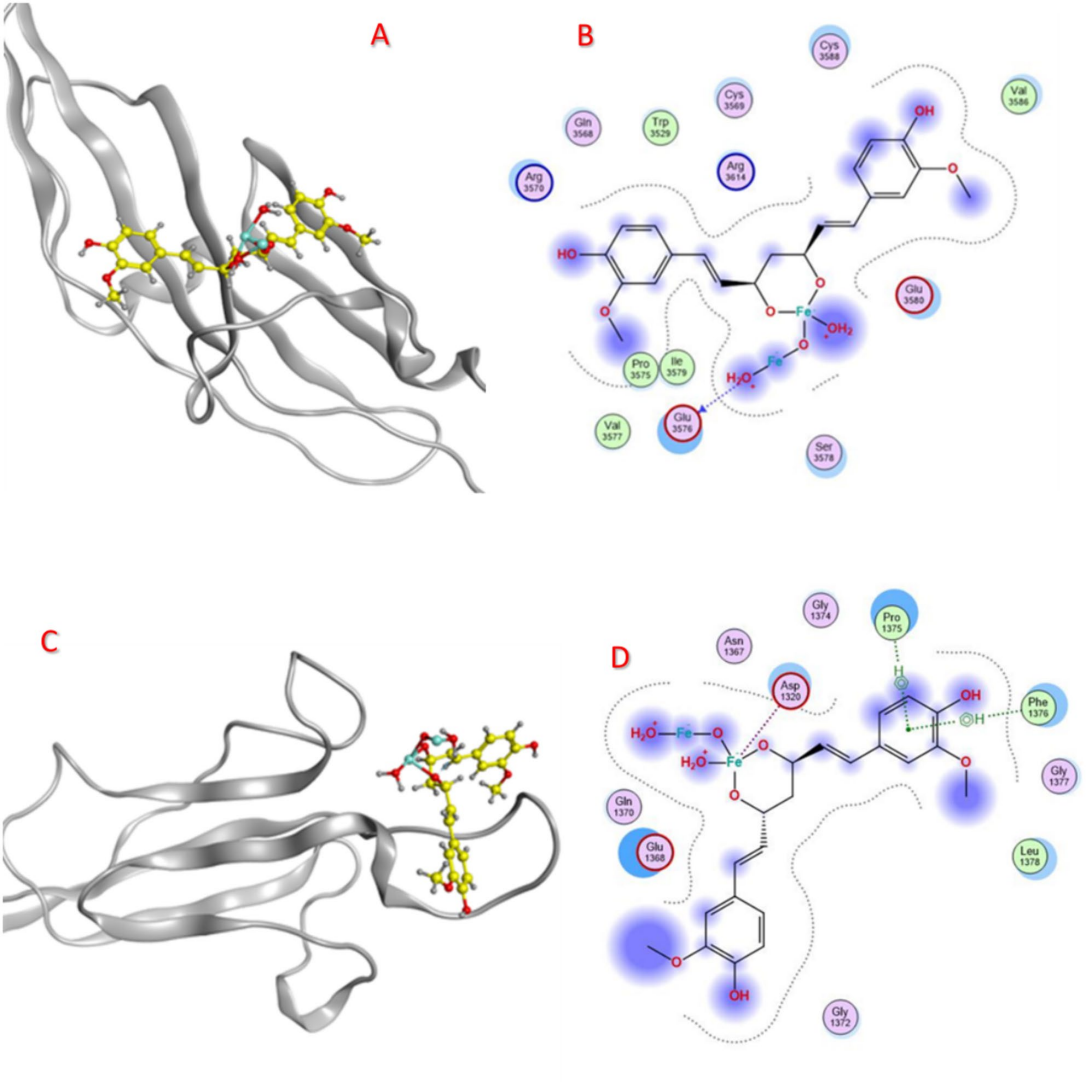
(**A**) cur-IONPs on the active site of muc 5AC, (**B**) interactions between cur-IONPs and muc 5AC, (**C**) cur-IONPs on muc2 active site, (**D**) interactions between cur-IONPs and muc 2.

The binding affinity scores of our selected coating agents and our cur-IONPs model to muc 5AC and muc 2 are consolidated in Table 2, and interactions are illustrated in Fig. 3. In molecular docking, a negative score signifies a favorable interaction, with more negative values indicating stronger binding. Therefore, the lowest (most negative) binding score suggests the highest likelihood of a stable and strong interaction between the protein and the ligand^15^.

**Table 2:** Cur-IONPs interactions with muc 5AC and muc2.

Muc 5AC								
ligand	S score	RMSD	Atoms donor	Atoms receptor	Bond type	E kcal	Distance	Residue
Cur-IONPs	− 6.0158	1.0807	O 56	O	H-donor	− 3.1	2.76	GLU 3576 (A)

Muc 2								
ligand	S score	RMSD	Atoms donor	Atoms receptor	Bond type	E kcal	Distance	Residue
Cur-IONPs	− 6.5806	2.6063	FE 50 6-ring 6-ring	OD2 CB CA	Metal pi-H pi-H	− 0.9 − 0.6 − 0.7	2.30 4.74 4.48	ASP 1320 (A) PRO 1375 (A) PHE 1376 (A)

### ADMET studies

As mentioned previously, ADMET analysis of this work was performed using SWISS-ADME and protox 3 servers by interring the canonical smile of our model, as shown in Table 3. The data for analysis were collected from both servers for further interpretation.

**Table 3:** Canonical smile and chemical formula of our cur-IONPs model.

IONPs-model	Canonical smiles	Formula
Cur-IONPs	O=[Fe]O[Fe]1(=O)OC(/C=C/c2ccc(c(c2)OC)O)CC(O1)/C=C/c1ccc(c(c1)OC)O	C21H22Fe2O9

### SWISS-ADME

**Fig. 4:**
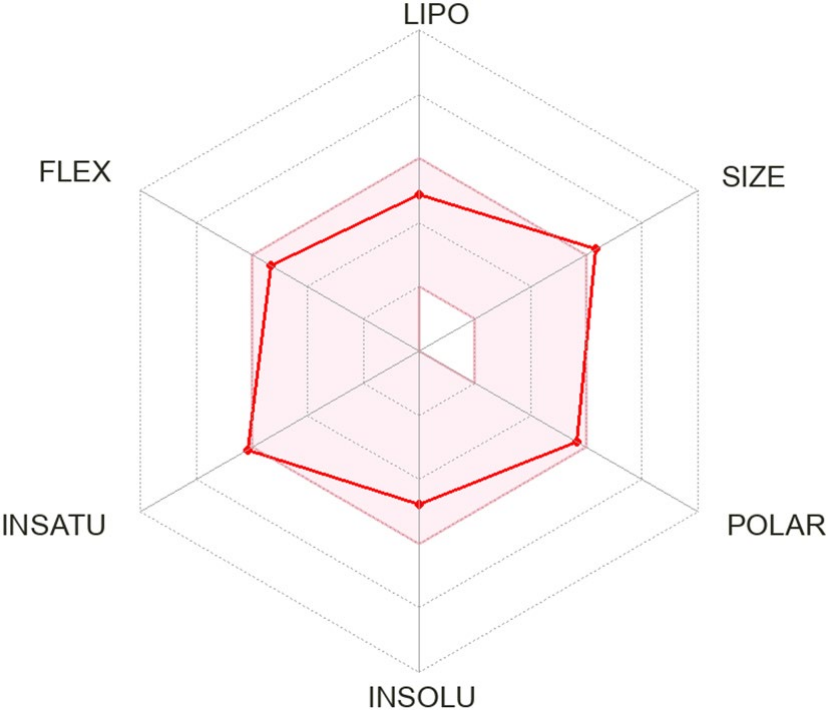
Bioavailability radar of cur-IONPs model.

We considered six physicochemical properties such as (solubility, size, lipophilicity, polarity, flexibility, and saturation) of cur-IONPs, that are visualized in the bioavailability radar (Fig. 4).

**Fig. 5:**
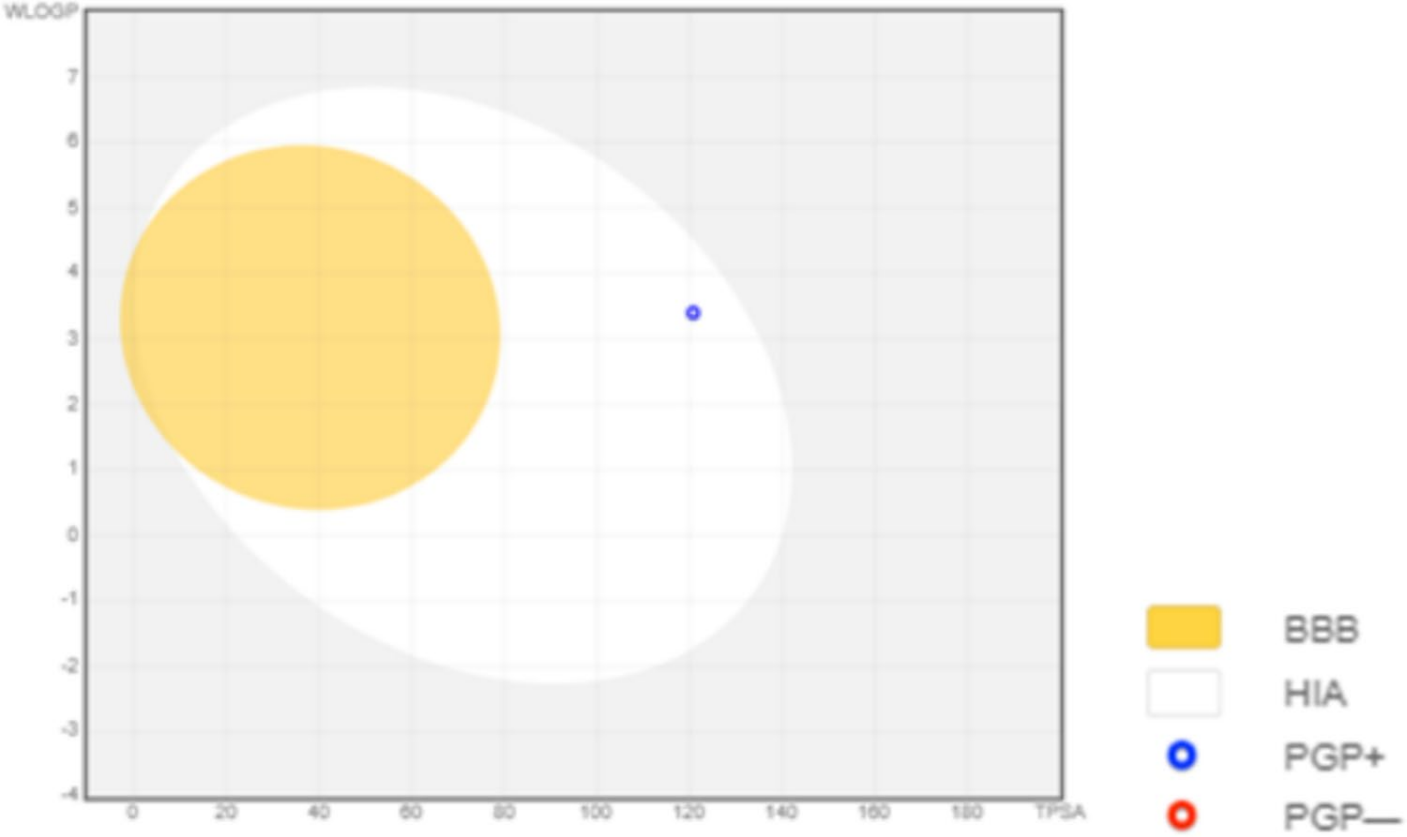
The boiled egg model for the modeled cur-IONPs.

Physicochemical properties, lipophilicity properties, water solubility, drug pharmacokinetics, and drug likeness properties are shown in Tables 4, 5, 6, 7, and 8, respectively. The boiled egg model for our compound is shown in Fig. 5.

**Table 4:** Physicochemical properties.

Compounds	MW	HA	AHA	RB	HBA	HBD	MR	TPSA
Cur-IONPs	530.08	32	12	8	9	2	104.92	120.75

**Table 5:** Lipophilicity characteristics.

compounds	iLOGP	XLOGP3	WLOGP	MLOGP	Silicos-IT Log P	Consensus Log P
Cur-IONPs	0	3.03	3.4	− 1.28	0.33	1.1

**Table 6:** Water solubility characteristics.

compounds	ESOL Log S	ESOL Solubility (mg/ml)	ESOL Solubility (mol/l)	ESOL Class	Ali Log S	Ali Solubility (mg/ml)	Ali Solubility (mol/l)	Ali Class
Cur-IONPs	− 4.78	8.70E−03	1.64E−05	Moderately soluble	− 5.23	3.11E−03	5.87E−06	Moderately soluble

Silicos-IT LogSw	Silicos-IT Solubility (mg/ml)	Silicos-IT Solubility (mol/l)	Silicos-IT class					
− 3.78	8.86E−02	1.67E−04	Soluble

**Table 7:** Drug pharmacokinetics.

compounds	GI absorption	BBB permeant	Pgp substrate	CYP1A2 inhibitor	CYP2C19 inhibitor	CYP2C9 inhibitor	CYP2D6 inhibitor	CYP3A4 inhibitor	log Kp (cm/s)
Cur-IONPs	High	No	Yes	No	No	No	No	Yes	− 7.38

**Table 8:** Drug likeness.

compounds	Lipinski #violations	Ghose #violations	Veber #violations	Egan #violations	Muegge #violations	Bioavailability Score
Cur-IONPs	1	1	0	0	0	0.55

### PRO-TOX 3

**Fig. 6:**
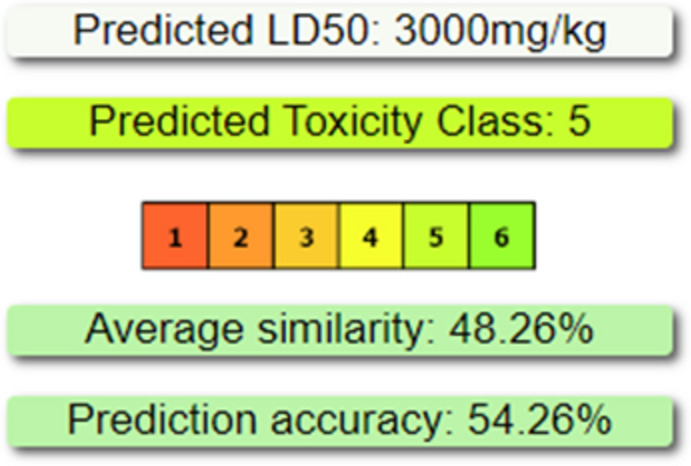
Oral toxicity prediction of modeled cur-IONPs.

The canonical smile of our modeled nanoparticles was interred in the Protox-3 server to analyze and predict oral acute toxicity, organ toxicity, toxicological and genotoxicological endpoints, and stress response pathways. Figure 6 indicates the predicted toxicity class of cur-IONPs and the rat's oral toxicity LD50 as mg/Kg. Regarding organ toxicity, all data for our compound are represented in detail in Table 9.

**Table 9:** Cur-IONPs protox prediction.

Classification	Target	Prediction	Probability
Organ toxicity	Hepatotoxicity	Inactive	0.71
Organ toxicity	Neurotoxicity	Inactive	0.86
Organ toxicity	Nephrotoxicity	Inactive	0.51
Organ toxicity	Respiratory toxicity	Inactive	0.51
Organ toxicity	Cardiotoxicity	Active	0.60
Toxicity endpoints	Carcinogenicity	Inactive	0.74
Toxicity endpoints	Immunotoxicity	Active	0.99
Toxicity endpoints	Mutagenicity	Inactive	0.70
Toxicity endpoints	Cytotoxicity	Inactive	0.76
Toxicity endpoints	BBB-barrier	Active	0.56
Toxicity endpoints	Clinical toxicity	Active	0.50
Toxicity endpoints	Nutritional toxicity	Inactive	0.75
Tox21-Stress response pathways	Nuclear factor (erythroid-derived 2)-like 2/antioxidant responsive element (nrf2/ARE)	Inactive	0.75
Tox21-Stress response pathways	Heat shock factor response element (HSE)	Inactive	0.53
Tox21-Stress response pathways	Mitochondrial Membrane Potential (MMP)	Inactive	0.68
Tox21-Stress response pathways	Phosphoprotein (Tumor Suppressor) p53	Inactive	0.84

### Molecular dynamic simulation

#### Cabs-Flex dynamic simulation

For the validation and estimation of protein–ligand complex stability, molecular dynamic simulation was performed for our cur-IONPs/proteins complexes.

**Fig. 7:**
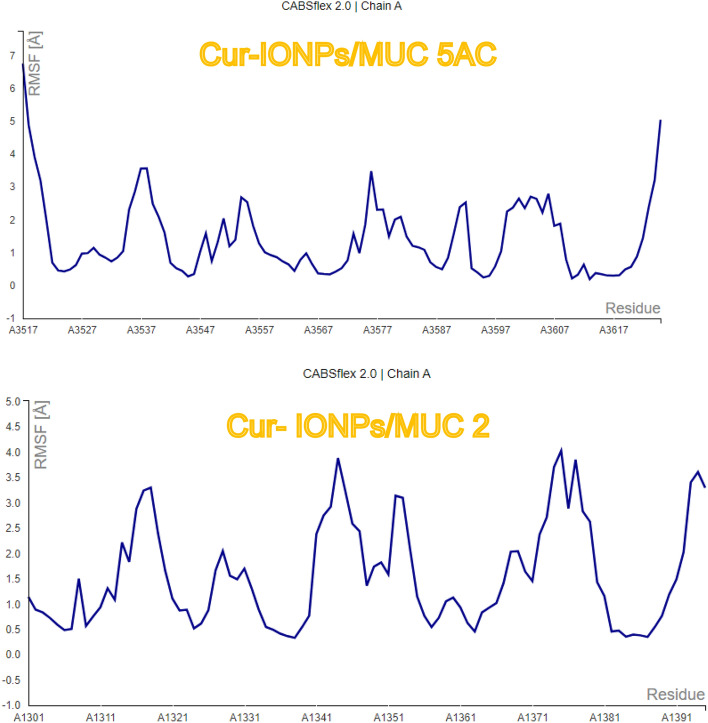
RMSF of cur-IONPs with muc 5AC and muc 2 proteins, using a CABSflex 2.0, through https://biocomp.chem.uw.edu.pl/CABSflex2.

CABS-FLEX was used to generate root mean square fluctuations (RMSF) profiles to assess the ligand-induced alteration in protein structure. The cabs-flex can generate RMSF profiles in addition to 10 models of structures after submitting the docked protein–ligand complex. Cabs flex results of RMSF are represented in Fig. 7.

#### IMODS dynamics

**Fig. 8:**
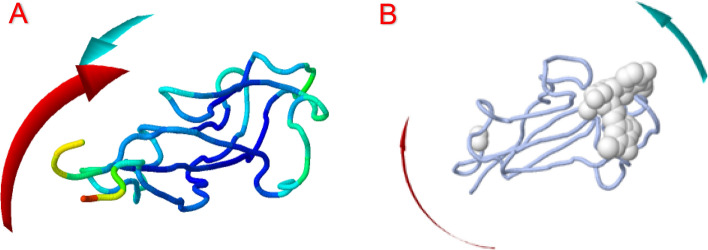
IMODS evaluated the molecular mobility of the docked ligand–protein complexes. The direction of motion is displayed with the two-colored affine arrows. The motion is greater if the arrow is longer, (**A**) cur-IONPs with muc 5AC, (**B**) cur-IONPs with muc2.

**Fig. 9:**
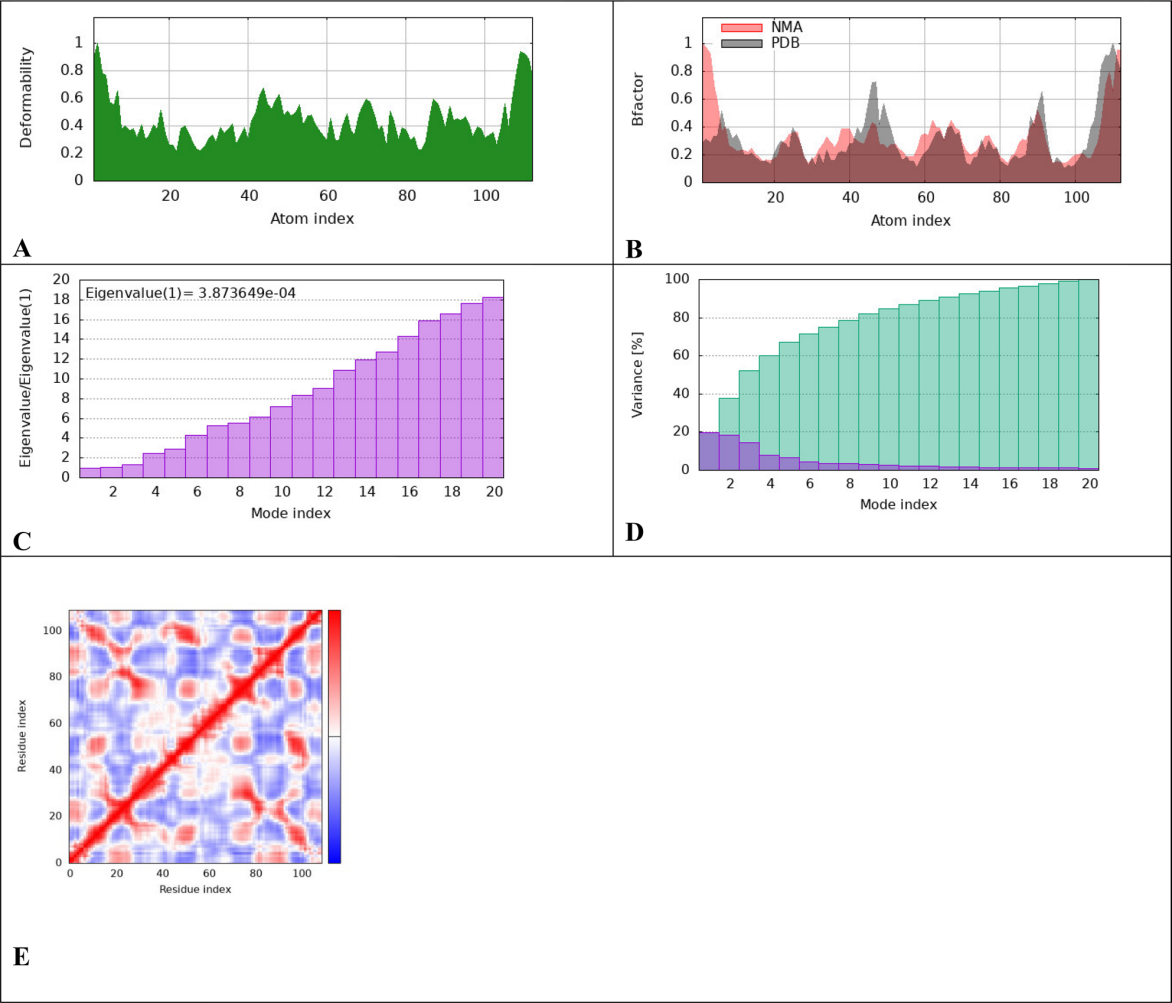
IMODS molecular dynamics simulations Outputs of cur-IONPs/Muc 5AC, Deformability graphs (**A**), B-factor plots (**B**), eigenvalue plots (**C**), variance map plots (**D**), correlation matrix plots (**E**).

**Fig. 10:**
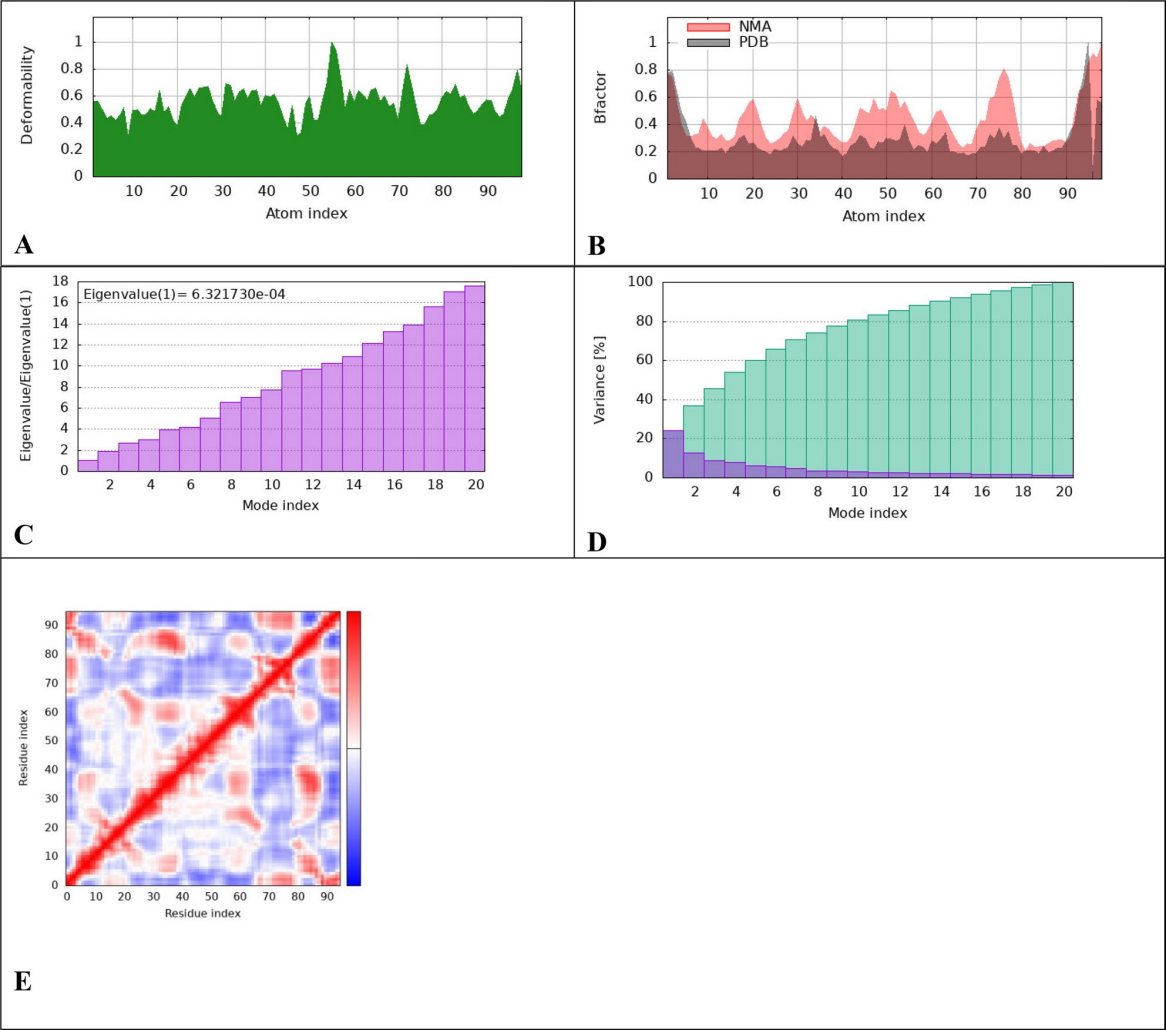
IMODS molecular dynamics simulations Outputs of cur-IONPs/Muc2, Deformability graphs (**A**), B-factor plots (**B**), eigenvalue plots (**C**), variance map plots (**D**), correlation matrix plots (**E**).

To evaluate the physical movement and stability of our ligand-proteins complexes, we performed MD simulation using IMODS. Normal mode analysis (NMA) was carried out to study the stability and slow dynamics of the cur-IONPs/proteins docked complexes. NMA of our docked complexes is illustrated in Figs. 8, 9, and 10.

## Discussion

The results of cur-IONP docking with muc5AC and muc2 are represented in Table 2. The cur-IONPs binding affinity was − 6.015 kcal/mol and − 6.58 kcal/mol for muc 5AC and muc 2, respectively. RMSD scores were 1.08A and 2.60A, as shown in Fig. 3. The cur-IONPs bounded to muc5AC with one H-bond with GLU 3576 (A) amino acid. In contrast, they bounded to muc 2 with three bonds, a metallic bond between the Fe atom and ASP 1320 (A), and two pi-H bonds with PRO 1375 (A) and PHE 1376 (A) residues. This could reflect the greater stability of cur-IONPs/muc2 than cur-IONPs/muc 5AC due to more bonds between the ligand and the protein. To investigate the toxicity of our model, its smiles code was delivered to SWISS-ADME and PRO-TOX-3 servers, six physicochemical properties were considered. From SWISS-ADME results, the bioavailability radar shows that our model falls entirely in the pink area as recommended, which means it can be regarded as oral bioavailable and drug-like. The pink area represents the optimal range for each property, as represented in detail in the SWISS-ADME user interface^16^.

Table 4 shows the physicochemical properties of the modeled cur-IONPs. In this section of the SWISS-ADME user interface, the general characteristic of our compound reveals that cur-IONPs molecular weight exceeds 500 Dalton. A molecular weight below 500 Dalton is a prime property of drug-likeness^22^.

The lipophilicity of our IONPs is shown in Table 5; SWISS-ADME provides five free models to evaluate the compound's lipophilicity characteristic, namely iLOGP, XLOGP3, WLOGP, MLOGP, and Silicos-IT Log P. The consensus Log P is the average of these predictions. The best range of Log P of a drug, according to the sources provided, is typically between 0 and 5, and 90% of marketed drugs are within this Log P range; having a Log P > 5 means that the drug is highly lipophilic, and it tends to accumulate in lipoidal areas of tissues and to have poor solubility. As presented in Table 5, we can see that cur-IONPs got a Log P score of 1.1, which is accepted, because this score means it will be optimal for oral administration due to the balance of solubility and permeability. (Table 6) shows that water solubility characteristics of modeled cur-IONPs were moderately soluble in adopted models.

Regarding drug pharmacokinetics, (Table 7) represents the pharmacokinetics parameters and bioavailability of our modeled IONPs and illustrates the boiled egg model for each of our compounds. According to boiled egg analysis in Fig. 5, cur-IONPs represented high HIA (human intestinal absorption).

P-glycoprotein (P-gp), also known as multidrug resistance protein 1 (MDR1), is an efflux transporter protein, Being a substrate or non-substrate of the permeability glycoprotein (P-gp) is crucial to appraising active efflux through biological membranes^17^, For instance, from the gastrointestinal wall to the lumen, despite cur-IONPs are represented as P-gp substrates it still has high HIA score, this means it may not being negatively affected by being a substrate to P-gp.

being P-gp substrate also could influence bioavailability and bio distribution of drug, it also could increase renal clearance^18^, this finding extremely stress on the importance of further in vivo studies focusing on dosage, efficacy, and rate of clearance of cur-IONPs. Despite that it was reported that coating IONPs with curcumin enhanced the bioavailability and clearance rate significantly as serum iron level began to decline slowly returning to its normal level after 3 weeks^19^.

Moreover, as illustrated in Table 7, cur-IONPs show an inhibition activity with one of the CYP 450 superfamily isoenzymes (CYP3A4). These enzymes are the key players in the detoxification and elimination through metabolic biotransformation. The inhibition activity of a drug against this category of enzymes could alter the clearance rate of the drug and increase its bioavailability and residency in the body. Furthermore, the best range of log Kp for a drug to be orally bioavailable and GI absorbable is between − 8 and − 1^20^. This means that cur-IONPs fall in the best range of this value for a designed drug for oral administration.

Regarding drug-likeness properties as shown in Table 8, the Lipinski filter (Pfizer) characterizes small molecules based on physicochemical property profiles such as MLOGP ≤ 4.15, Molecular Weight (MW) less than 500, NH or OH ≤ 5, N or O ≤ 10,^21^. Table 8 shows a drug-likeness rule score according to Lipinski Ghose, Veber, Egan, and Muegge. We found that cur-IONPs met four of the five drug-likeness rules, except the Ghose rule, with one violation due to its high molecular weight MW > 480. Despite the molecule fitting Lipinski's rule, it shows one violation due to the high MW, MW > 500, however our molecule has MW = 530 g/mol which exceeds the desired upper limit of MW slightly, it is not an absolute requirement for efficacy or approval as according to the rule of five a drug can have one violation of these criteria and still be considered likely to be an effective oral drug, in fact many FDA approved drugs exceeded 500 g/mol. Finally, regarding bioavailability, cur-IONPs get a score of 0.55, which is considered good and accepted^22^. Our SWISS-ADME results conclude that cur-IONP is a good candidate for oral administration in treating IDA and deserves focus and further investigations.

Protox-3 server analyzes and predicts oral acute toxicity, organ toxicity, toxicological and genotoxicological endpoints, and stress response pathways. Figure 6 indicates the rat's oral toxicity lethal dose of 50 (LD_50_) as mg/Kg, cur-IONPs obtained LD_50_ value (3000 mg/Kg), which reflects the safety of cur-IONPs if it is administered orally^23^. The oral toxicity assessment of cur-IONPs indicates that they are generally safe when administered orally. The study on male BALB/c mice, where 6 doses of 5 mg/kg Cur-IONPs were given on alternating days for two weeks, showed promising results regarding toxicity and biodistribution. Additionally, another study reported the long-term safety and stability of cur-IONPs after a single dose administration in mice^19^.

The predicted toxicity class of cur-IONPs was 5, respectively, as shown in Fig. 6. Regarding organ toxicity, all data are represented in detail for our compound in Table 9. Cur-IONPs exhibit hepatotoxic-inactive or non-hepatotoxic with a probability score of 0.71, cardiotoxicity, immune toxicity, and clinical toxicity with prediction scores of 0.60, 0.99, and 0.50, respectively. Cur-IONPs were investigated in vitro and in vivo and many studies assure its safety as reported in a study involving male BALB/c mice treated with multiple doses of Cur-IONPs showed no significant differences in liver enzyme levels (AST, ALT) compared to control groups, in the same study The biodistribution of Cur-IONPs revealed that they predominantly accumulated in the liver, spleen, and brain. Notably, histopathological examinations showed no abnormalities in these organs, indicating that the presence of Cur-IONPs did not lead to visible tissue damage^19^. Regarding suspected cardio toxicity, immune toxicity, and clinical toxicity of our molecule, to the best of our knowledge we found no previous study reported any related observations, on contrary cur-IONPs coating IONPs with curcumin was reported to enhance biocompatibility and safety in comparison to uncoated IONPs^19,24,25^.

This suggests that Cur-IONPs do not induce notable organ toxicity under the tested conditions and more investigations could be very beneficial in this point.

Furthermore, it is predicted to be able to cross the blood–brain barrier with prediction scores of 0.56, this comes in agreement with previous studies which reported the ability of cur-IONPs to cross BBB^19^. Regarding genotoxicity, our compound is not suspected to be carcinogenic, mutagenic, or cytotoxic, and it is predicted not to activate stress pathways as presented in the Table 9, in fact the role of curcumin as antioxidant^26^, anticancer^27,28^, and anti-inflammatory agent^29^ is well known. Based on the results, cur-IONPs show a favorable safety profile and promising results for further application as therapeutic nanoparticles. From our docking results, in addition to ADMET studies, we conclude that cur-IONPs would be potential for further investigations, so cur-IONPs/protein complexes interred to molecular dynamic simulation to assess the stability of the complex in more reliable ways, mimicking the real interaction environment in our body.

To assess the ligand-induced alteration in protein structure, CABS-FLEX was used to generate root mean square fluctuations (RMSF) profiles as illustrated in Fig. 7. CABS-FLEX can generate RMSF profiles in addition to 10 models of structures after submitting the docked protein–ligand complex. RMSF value is usually calculated to indicate the stability of the complex, and the best RMSF value should be 1–3 A for optimum stable binding conditions. CABS-FLEX can generate RMSF from 10 ns simulations of all proteins/ligand complexes. Cur-IONPs/muc 5AC showed a max RMSF of 8.19 at residue 3516, a min RMSF was 0.195 at residue 3613, and an average RMSF was 1.49, while cur-IONPs/muc 2 showed a max RMSF of 4.028 at residue 1375, min RMSF of 0.333 at residue 1338, and average RMSF equals 1.567. Our results from the CABS-FLEX dynamic simulation showed that cur-IONPs/protein complexes are stable. Most complex residues show RMSF values below 3A, which is the main indicator of structure stability^30^. A lower value of RMSF specifies restricted movements from the average position throughout the simulation, while a higher value of RMSF demonstrates more movement flexibility^31^.

To evaluate the physical movement and stability of our ligand-proteins complexes, we performed MD simulation using IMODS. (NMA) normal mode analysis was conducted to investigate the stability of our docked complexes. NMA of our docked complexes is illustrated in Figs. 9 and 10. The deformability and B-factor of complexes display the peaks corresponding to the regions with deformability in the protein, the highest peaks the highest deformability. The docked complexes' significant mobility was shown by the NMA analysis, demonstrating the structural flexibility of the proteins with cur-IONPs. Furthermore, the deformability of proteins is demonstrated by our data; most of the proteins had several peaks that showed deformability. The mobility of the protein is related to the B-factor, and the energy required to deform the structure is directly correlated with the eigenvalues produced for the docked proteins. It represents the protein–ligand complex's motion stiffness. The complex has stronger stability and easier deformability with a lower eigenvalue.

The bound proteins’ MD analysis showed that the complexes have a notable degree of deformability. Additionally, the cur-IONPs/muc 5AC and cur-IONPs/muc 2 complexes had eigenvalues of 3.87 e−04 and 6.32 e−04, respectively, demonstrating the good flexibility and stability of the molecular motion of the docked complexes. The complexes' covariance matrix shows the correlations between the residues in each complex. In the matrix, the red color denotes a reasonable degree of correlation between residues, while the white color denotes uncorrelated motion. Based on the plausible interactions of the selected proteins with cur-IONPs, we speculate that it can serve as a potential drug candidate for a highly bioavailable and long-resident IDA treatment. More in-vitro and in-vivo studies should be conducted to clearly reveal the toxicity of cur-IONPs and assess their efficacy as a potential drug for IDA.

## Conclusion

In silico studies on our model of cur-IONPs reveal that it has the potential to be well investigated in vitro and in vivo as a good candidate for IDA treatment. Cur-IONPs exhibit a mucoadhesive behavior with considered affinities towards muc 5AC and muc 2 proteins in GIT, which are supposed to positively impact the drug's bioavailability, absorption, and residency. In silico toxicity predictions on cur-IONPs reveal their safety and drug-likeness. Furthermore, molecular dynamic simulation studies approved the stability and flexibility of protein–ligand complexes.

## Data Availability

The dataset generated and/or analyzed during the current study are available from the corresponding author on reasonable request.
